# Anterior Mandibular Lingual Foramina: An In Vivo Investigation

**DOI:** 10.1155/2014/906348

**Published:** 2014-08-25

**Authors:** Sara Bernardi, Claudio Rastelli, Cinzia Leuter, Roberto Gatto, Maria Adelaide Continenza

**Affiliations:** Department of Life, Health & Environmental Sciences, University of L'Aquila, Via Vetoio 2, Frazione Coppito, 67100 L'Aquila, Italy

## Abstract

In descriptions of surgical procedures in mandible, often there is no mention of an anatomical variance, the genial spinal foramina, where nerves and vessels go through. Aim of this study is to investigate frequency, shape, and dimensions of these foramina. 56 computed tomography dentascans were analyzed with an implant planning software. The considered parameters were frequency, number, position, diameters, and length of canals; the collected data were inserted in a spreadsheet and statistically analyzed; therefore, they were compared with those found in the literature. The measurements agree with the ones found in earlier studies, except for the length of the inferior spinal canals, which resulted lesser than that found in the literature. The frequency of the inferior spinal foramina, the data related to the inferior spinal foramina diameter (cross scan), and the measurements related to the superior spinal foramina diameter (axial scan) resulted to be major compared to those reported in literature. These obtained results are clinically interesting because an implant planning software has been employed, daily used by operators, and that permits in vivo investigations. Furthermore, due to the possibility of hemorrhagic accidents in this mandibular region, these data are particularly interesting for all of the operators who make interventions in this area.

## 1. Introduction

The rate of implant therapy with the aim to restore a correct functionality of oral apparatus involves a number of evaluations to prevent the possibility of neurovascular accidents and complications, which could happen during this type of surgical procedure.

This foreword explains the increased interest in studying anatomy of orofacial district and the development of software dedicated to analysis of radiological images (particularly computed tomography dentascan), with the purpose to find out as much as possible information to plan correctly the surgical intervention.

In descriptions of these surgical procedures in inferior jaw, the anterior lingual foramina and related vessels and nerves are not often mentioned.

These foramina contain the destination of branches of lingual artery vein and nerve. They penetrate the cortical side of mandible, in the incisors' region, near the mental spines.

The first authors who made a deep illustration of this anatomical structure were Bertelli [1] and then, in 1937, Ennis [2]. This last one named them as “interspinous foramina,” changing the earlier and ambiguous denomination “median mental foramina” [1].

After Ennis, many authors were concerned about these formations and, among them, McDonnell et al. in 1994 [3] underlined the presence of other foramina which also contain the terminal branches of sublingual artery.

The most recent studies with both anatomical dissection [4, 5] on inferior jaw and radiological evaluation with CT dentascan method [6–8] have defined and catalogued the presence of these foramina.

**Figure 1:**
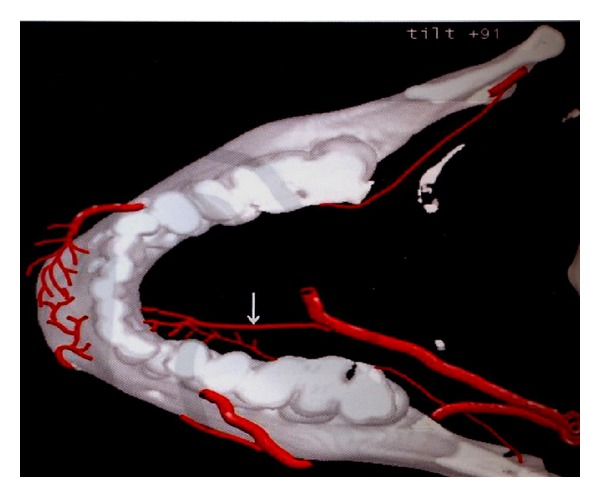
Reconstruction of anatomical structures cited in the text (the white arrow points at the sublingual artery of which ramifications penetrate the mandibular cortical bone), from the book “Diagnostica per Immagini in Implantologia Orale,” Diotallevi P., Moglioni E., Casa editrice CIC.

**Figure 2:**
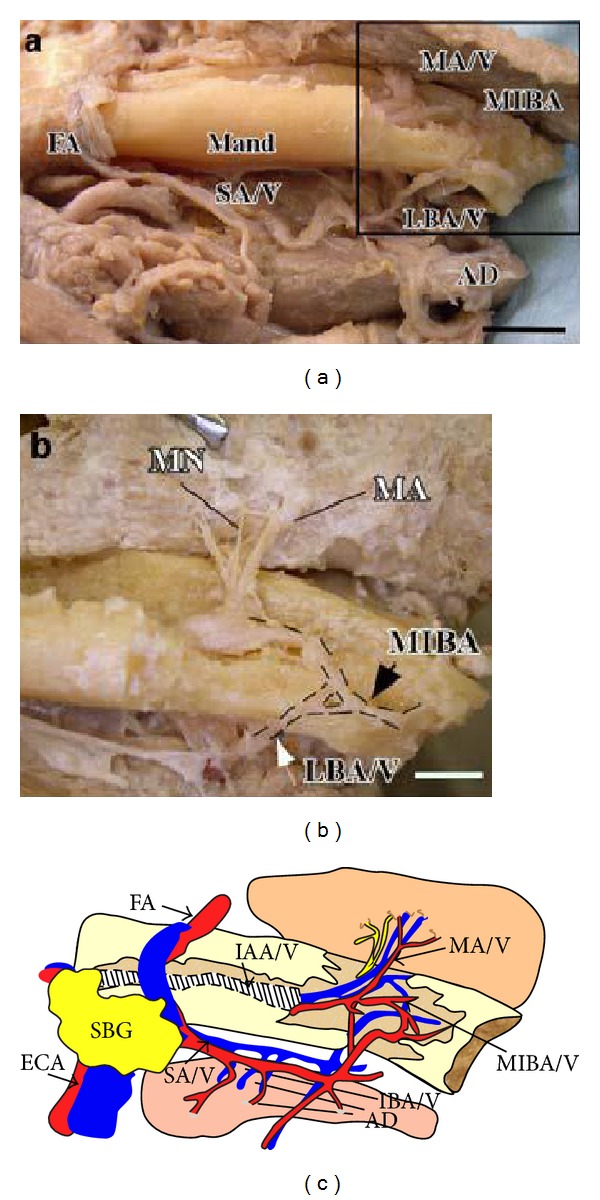
(a) Dissection of the IAA and its terminal branches. In this case, the IAA terminates by dividing into a mental incisive branch and a lingual branch, which communicates with the SA in the submandibular triangle. Before passing through the LF, the lingual branch also connects with the incisive branch in the anterior region (arrows). (a) Anteroinferior view of the mental region of the mandible. (b) Lateral view of the mental region of the mandible (see square of (a)). (c) Schematic illustration of the dissection shown in (a). AD: anterior belly of the digastric muscle; ECA: external carotid artery; FA: facial artery; IAA/V: inferior alveolar artery and vein; LBA/V: lingual branch of artery and the vein; Mand: mandible; MA/V: mental artery and vein; MIBA/V: mental incisive branch of artery and vein; MN: mental nerve; SA/V: submental artery and vein; SBG: submandibular gland. Bar 1 cm (from [13]).

They established a classification as well: the foramina located up the genial spines are denoted as superior genial spinal foramina and involve the branches of lingual artery, vein, and nerve. The foramina located down the genial spines are denoted as inferior genial spinal foramina and contain sometimes the branches of sublingual artery and vein and sometimes the submental vessels and branches of mylohyoid nerve (Figures 1 and 2).

These neurovascular formations can be involved in implant surgery in this region [9].

Besides the anatomical information, these studies have the purpose to prevent the hemorrhagic episodes in sublingual region, which are reported in the literature [10–12], air obstruction due to the formation of hematoma, consequence of the cortical perforation, and/or the laceration of periosteum with the involvement of the cited vessels.

Aim of this study is to investigate the frequency, the shape, and the dimension of anterior mandibular lingual foramina and their canals.

## 2. Materials and Methods

Fifty-six mandibular CT dentascans from an Italian central region (Abruzzo) population were collected, with patients' consensus; the age average of patients was between 16 and 80 years.

The radiological images were labeled with a code and analyzed with an implant planning software “Micerium Implant Planning” (MIP in the text). For each subject was made a table where measured data were inserted.

The parameters described were the following:

1. frequency;
2. number;
3. localization;
4. length of related canals;
5. diameters.

For what concerns the last parameter, the measures have been taken both on axial sections and on cross sections, on lingual and labial sides.

**Figure 3:**
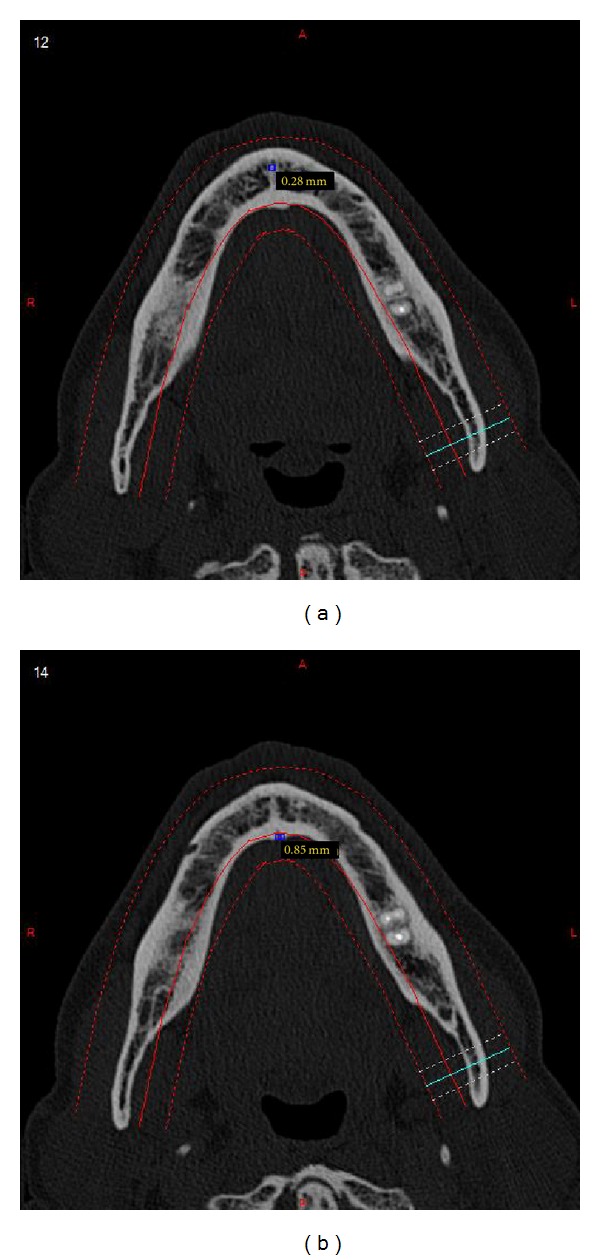
(a) Axial section with measure of foramina diameter, labial side. (b) Axial section with measure of foramina diameter, lingual side.

**Figure 4:**
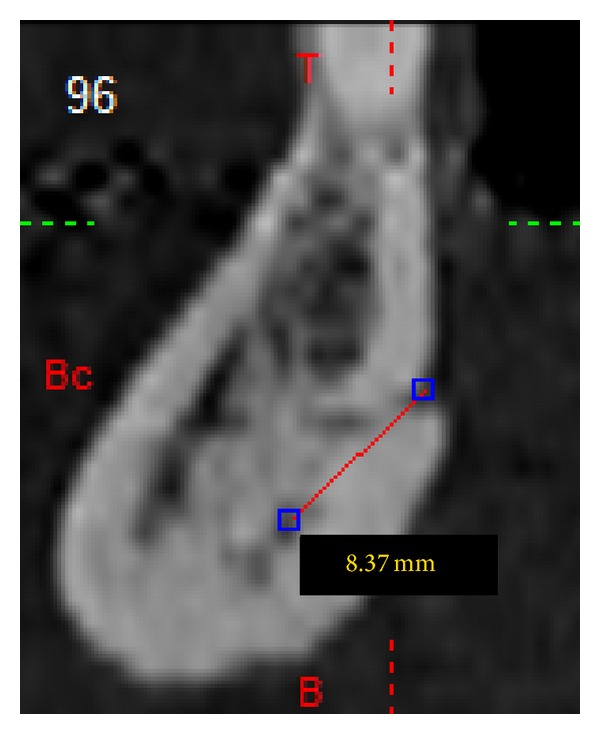
Cross section with measurement of superior canal.

**Figure 5:**
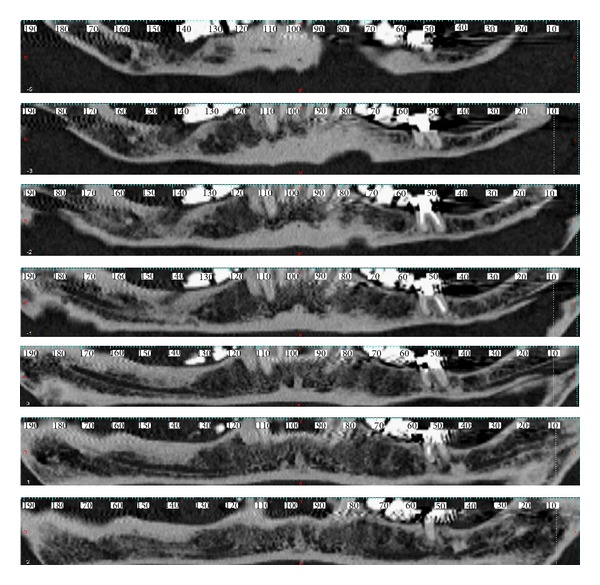
Panorex sequences.

**Figure 6:**
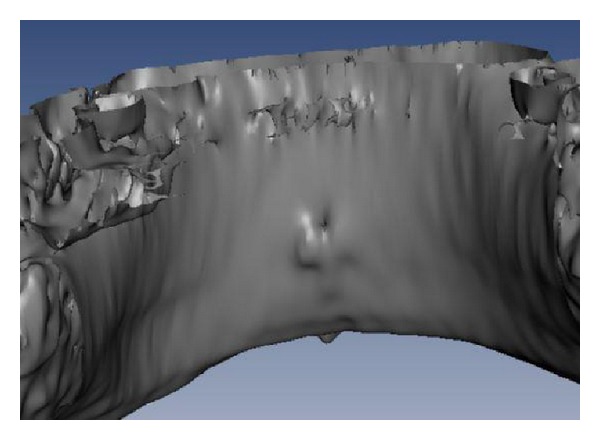
3D reconstruction.

After DICOM sequences had been imported on MIP program, axial sections (Figures 3(a) and 3(b)), cross sections (Figure 4), Panorex sequences (Figure 5), and 3D reconstructions (Figure 6) were obtained and analyzed.

The data were statistically analyzed with descriptive statistics methods, evaluating the parameters with mean and standard deviation functions; Student's *t*-test was used to validate and to compare the collected data with literature ones. All the statistical analyses were performed using the software SPSS version 19.

## 3. Results

Among the 56 inferior CT dentascans investigated, 43 (75%) had at least one foramen on the lingual mandibular midline. Between these 43, 27 (62%) samples resulted having the foramen up to the genial spines and 6 (13%) down the genial spines.

Furthermore, amid these 43 samples, 10 (23%) had two foramina, one superior and one inferior to the spines. Only one mandible showed two foramina, both down the mental spines. Student's *t*-test validated those results (*P* value < 0,00001).

From comparison between these data and those in literature (Table 1), it came out that they agree perfectly, although the percentage of presence in this study is minor, considering the absolute value, compared with the ones reported in the earlier studies.

Another datum that disagrees (absolute value consideration) with the literature is the frequency of inferior genial spinal foramina, resulting minor in this study. These differences were considered statistically significative according to the chi-square test (*P* < 0.05) (Table 2).

For what concerns the other parameters, the length of related intrabony canals resulted in superior ones being meanly longer than inferior ones (Table 3).

The evaluations of the diameters showed that the diameter of superior genial spinal foramina, lingual side, on axial sections, was meanly 0,83 ± 0,28 mm; meanwhile, on cross sections it was 1,24 ± 0,32 mm.

The diameter of inferior genial spinal foramina, lingual side, on axial sections, resulted as 0,97 ± 0,38 mm, and instead the mean value of cross section was 0,92 ± 0,26 mm.

The diameter of superior genial spinal foramina, labial side, on axial sections, resulted to be 0,48 ± 0,24 mm, whereas on cross sections it was 0,63 ± 0,30 mm; the diameter of inferior genial spinal foramina, labial side, on axial sections, resulted as 0,54 ± 0,23 mm, and instead the mean value of cross section was 0,53 ± 0,17 mm.

As for all these parameters, the performed Student's *t*-tests validated all the data as the *P* value resulted < 0,0001.

The differences between the two foramina resulted as not statistically significative, except for the intrabony canal length.

From literature data comparison, it came out that some measurements such as the diameter of superior genial spinal foramina, lingual side, axial sections, and inferior genial spinal foramina lingual side cross sections resulted to be major compared to the data found in earlier studies and the differences could be considered statistically significative (*P* < 0.05).

The morphometric analysis revealed that the foramina on lingual side have meanly an ovalar shape: the superior ones have the major axis located vertically and the inferior ones have the major axis located horizontally.

The foramina on labial side resulted smaller than the foramina on lingual side, with an ovalar shape in the superior ones and with a circular shape in the inferior ones.

## 4. Discussion

Many studies on accessory mandibular foramina and related bony canals have been found in the literature. Some of them [3, 16, 17] reported data obtained with anatomical dissection method. Other authors [5] showed results obtained with traditional radiological method (2D) like orthopantomography; others yet [7, 8, 14, 15, 18] have used tridimensional radiological method (CT dentascan, CBCT).

In all of these studies, the reported frequency of foramina is high, and in some of them, both on radiological scans and on anatomical dissections, respectively, it reaches a frequency of 80% and 92%.

These results were confirmed using CBCT method, founding a frequency of 100% [7, 14].

From the dissectors studies [3, 13, 19, 20] the neurovascular way of the connected structures has also been cleared (branches of sublingual and submental artery, mylohyoid nerve variants [21]).

The sublingual artery rises near the anterior board of hyoglossus muscle. The vessel proceeds horizontally between the mylohyoid muscle, externally, and the geniohyoid muscle, internally.

It goes then along the medial face of sublingual gland; it makes an anastomosis with the contralateral one.

It perforates the cortical bone on lingual side, passing through the lingual foramina and anastomoses with the central alveolar vessels [4].

The submental artery is an offshoot of facial artery; it starts near the submandibular grand, goes internally, along the inferior face of mylohyoid muscle with the mylohyoid nerve, and ends in mental region, making anastomosis with the ramifications of anterior alveolar artery.

The sublingual and submental arteries go parallel along the surface of mylohyoid muscle: the first one medially and superiorly to the surface muscle and the second one laterally and inferiorly to the surface muscle.

For what concerns the mylohyoid nerve, the dissector studies confirmed the variant way reported early in the previous studies. These authors reported in 50% of cases a supplementary branch that penetrates in the mandible through the accessory foramina [22].

All of these authors give also the measurements of the foramina diameters, even if often they do not specify if they are relating to superior or inferior foramina, or they give a specific description just for one type of diameter (pointing out the major value as the long axis and the minor value as the short axis).

They also underline the importance of the relationship between the diameter and the size of vessels and that the vessels with a diameter less than 1 mm are a potential hemorrhagic index risk.

The results that came out from this study give morphological information on spinal lingual foramina and related intrabony canals. They agree both with the most recent study [4, 15] and with the earlier ones, even if four parameters deviate from them: (1) the effective presence, (2) the presence of inferior foramina, (3) the diameters, and (4) the length of canals. These variances are probably due to both the number of specimens and the method to obtain the data.

In this study the employment of an implant planning software to analyze CT dentascan images gave precise information and details about shape of these foramina and about the course of anatomical structures that go through them.

### 4.1. Strengths and Limitations

The main limitation of this study is the limited number of the samples; by the way, the study gives information more detailed about the morphology of this particular anatomical variance. Furthermore, the use of an implant planning software planning on computed tomography dentascan can integrate the information about morphological details.

## 5. Conclusions

The results obtained in this study are clinically interesting because an implant planning software has been employed, daily used by operators, and that permits in vivo investigations. Furthermore, due to the possibility of hemorrhagic accidents in the anterior region of the mandible, these data are particularly interesting for all of operators who make interventions in this area.

## Figures and Tables

**Table 1:** Frequencies of presence parameter.

Total	Present (%)	Literature data (%)
57	75	98∗/100∗∗

∗From lingual foramina on the mandibular midline revisited: a macroanatomical study; Liang et al. [4].

∗∗From macroanatomic and radiologic characteristics of the superior genial spinal foramen and its bony canal; Vandewalle et al. [5].

**Table 2:** Frequencies of position parameter.

Foramen	Present (%)	Literature data (%)
Superior	62	62∗/63,3∗∗
Inferior	13	38∗/13,34∗∗
Two foramina	23	22∗/23,3∗∗/27,1∗∗∗

∗From lingual foramina on the mandibular midline revisited: a macroanatomical study; Liang et al. [4].

∗∗From cone beam computed tomography observations of the lingual foramina and their bony canals in the median region of the mandible; Babiuc et al. [14].

∗∗∗From computed tomographic diagnosis and localization of bone canals in the mandibular interforaminal region for prevention of bleeding complications during implant surgery; Tepper et al. [15].

**Table 3:** Means and standard deviations of diameters and length of intrabony canals parameter.

		S1	S2	Literature S1∗	Literature S2∗
Lingual side diameter cross section	Mean DS	0,8 mm, 0,17	1,09 mm 0,4	0,9 mm∗∗ 0,4	0,8 mm∗∗ 0,4
Lingual side diameter axial section	Mean DS	1,24 mm 0,29	0,92 mm 0,2	0,7 mm∗∗∗ 0,2	Absent
Labial side diameter cross section	Mean DS	0,42 mm 0,18	0,56 mm 0,19	0,4 mm∗∗ 0,3	0,5 mm∗∗ 0,3
Labial side diameter axial section	Mean DS	0,65 mm 0,20	0,58 mm 0,18	Absent	Absent
Length of canals	Mean DS	6,35 mm 2,28	4,38 mm 1,43	6,8 mm∗∗ 2,3	6,1 mm∗∗ 2,6

∗S1 indicates the superior genial spinal foramina and S2 denotes the inferior genial spinal foramina.

∗∗From lingual foramina on the mandibular midline revisited: a macroanatomical study; Liang et al. [4].

∗∗∗From macroanatomic and radiologic characteristics of the superior genial spinal foramen and its bony canal; Vandewalle et al. [5].
